# Elaboration, validation and reliability of the safety protocol for pediatric thirst management*

**DOI:** 10.1590/1518-8345.3333.3321

**Published:** 2020-07-15

**Authors:** Isadora Pierotti, Leonel Alves do Nascimento, Edilaine Giovanini Rossetto, Rejane Kiyomi Furuya, Lígia Fahl Fonseca

**Affiliations:** 1 Universidade Estadual de Londrina, Centro de Ciências da Saúde, Londrina, PR, Brazil.; 2 Scholarship holder at the Fundação Araucária de Apoio ao Desenvolvimento Científico e Tecnológico do Estado do Paraná (FA), Brazil.; 3 Hospital Dr. Anisio Figueiredo, Zona Norte de Londrina, Londrina, PR, Brazil.; 4 Instituto Federal do Paraná, Campus Londrina, Londrina, PR, Brazil.

**Keywords:** Thirst, Operating Room Nursing, Recovery Room, Pediatrics, Clinical Protocols, Validation Studies, Sede, Enfermagem de Centro Cirúrgico, Sala de Recuperação, Pediatria, Protocolos Clínicos, Estudos de Validação, Sed, Enfermería de Quirófano, Sala de Recuperación, Pediatría, Protocolos Clínicos, Estudios de Validación

## Abstract

**Objective:**

to elaborate, validate and evaluate the reliability of the Safety Protocol for Pediatric Thirst Management in the immediate postoperative period.

**Method:**

methodological quantitative research, based on the assumptions on measurement instrument development. The protocol was elaborated after literature review, interview with specialists and observation of the child’s anesthetic recovery. The judges performed theoretical validation through apparent, semantic and content analysis. Content Validity Index was calculated for content validation, whose minimum established concordance was 0.80. Protocol’s reliability was evaluated in children between three and 12 years old in the Post Anesthesia Care Unit.

**Results:**

in its final version, the protocol consisted of five evaluation criteria: level of consciousness, movement, airway protection, breathing pattern and nausea and vomiting. It presented easy comprehension and relevant content, and all indexes exceeded the minimum agreement of 0.80. Pairs of nurses applied the protocol 116 times to 58 children, resulting in a high reliability index (*kappa* general = 0.98)

**Conclusion:**

the unprecedented protocol developed is valid and is a useful tool for use in anesthetic recovery, aiming to assess safety for reducing the thirst of infant patients.

## Abstract (pt)

**Objetivo:**

elaborar, validar e avaliar a fidedignidade do Protocolo de Segurança para o Manejo da Sede Pediátrica no pós-operatório imediato.

**Método:**

pesquisa metodológica, quantitativa, baseada nos pressupostos para elaboração de instrumentos de mensuração. Elaborou-se o protocolo após revisão de literatura, entrevista com especialistas e observação da recuperação anestésica da criança. Juízes realizaram a validação teórica por meio de análise aparente, semântica e de conteúdo. Calculou-se o *Content Validity Index* na validação de conteúdo, cuja concordância mínima estabelecida foi de 0,80. Avaliou-se a fidedignidade do protocolo em crianças entre três e 12 anos em sala de recuperação anestésica.

**Resultados:**

em sua versão final, o protocolo foi constituído por cinco critérios de avaliação: nível de consciência, movimentação, proteção de vias áreas, padrão respiratório e náusea e vômito. Apresentou fácil compreensão e conteúdo pertinente e relevante, e todos os índices superaram a concordância mínima de 0,80. Duplas de enfermeiros aplicaram o protocolo 116 vezes em 58 crianças, resultando em elevado índice de fidedignidade (*kappa* geral = 0,98)

**Conclusão:**

o protocolo inédito elaborado tem validade e é ferramenta útil para utilização na recuperação anestésica, visando avaliar a segurança para a redução da sede do paciente infantil.

## Abstract (es)

**Objetivo:**

elaborar, validar y evaluar la confiabilidad del Protocolo de Seguridad para el Manejo de la Sed Pediátrica en el postoperatorio inmediato.

**Método:**

investigación cuantitativa, metodológica, basada en los presupuestos para la elaboración de instrumentos de medición. El protocolo se elaboró después de la revisión de la literatura, entrevista con especialistas y observación de la recuperación anestésica del niño. Los jueces realizaron la validación teórica a través del análisis aparente, semántico y de contenido. El *Content Validity Index* se calculó para la validación de contenido, cuya concordancia mínima fue de 0,80. La fiabilidad del protocolo se evaluó en niños entre tres y 12 años en la sala de recuperación anestésica.

**Resultados:**

en su versión final, el protocolo consistió en cinco criterios de evaluación: nivel de conciencia, movimiento, protección de las vías respiratorias, patrón respiratorio y náuseas y vómitos. Presentó fácil comprensión, con contenido pertinente y relevante, siendo que todos los índices excedían la concordancia mínima de 0,80. Parejas de enfermeros aplicaron el protocolo 116 veces a 58 niños, lo que resultó en un índice de alta confiabilidad (*kappa* general = 0,98).

**Conclusión:**

el protocolo desarrollado, que reviste carácter inédito, es válido y puede considerarse como herramienta útil para su uso en la recuperación anestésica, con el objetivo de evaluar la seguridad para reducción de la sed del paciente infantil.

## Introduction

The perioperative period brings innumerable coping challenges for the child. In the preoperative period, preparations inherent to the procedure, such as fasting, bring anxiety and discomfort^(^ 1 ^-^ 3 ^)^. Fasting is indicated in order to avoid adverse events such as bronchoaspiration for gastric contents^(^ 2 ^)^. Although its indication is recognized and the literature currently recommends reduced fasting times^(^ 1 ^-^ 6 ^)^, excessive periods are identified in practice^(^ 2 ^,^ 4 ^)^. Recent evidence shows that abbreviating the fasting time not increase adverse events incidence^(^ 1 ^-^ 6 ^)^.

Fasting is extended to the immediate postoperative period (IPP) and fluids are usually released in the first three hours for most children^(^ 7 ^)^. However, a clinical trial revealed that fluid intake even more precociously in the Post Anesthesia Care Unit (PACU) did not increase the incidence of nausea and vomiting^(^ 8 ^)^. The benefits of early fluid release in the IPP are: More parental satisfaction, happier and less uncomfortable children with pain, reduced use of medication for nausea, reduced length of stay in PACU, and reduced thirst^(^ 8 ^-^ 10 ^)^.

Anesthetic recovery is characterized by the return of consciousness and during awakening, the child may experience pain, being confused and agitation. Thirst also influences the child’s mode of awakening and recovering from anesthesia, being one of the factors responsible for the anguish they experience in this period^(^ 8 ^-^ 9 ^,^ 11 ^-^ 13 ^)^.

The surgical child is at high risk for developing thirst due to hydroelectrolytic imbalance, endotracheal intubation, use of medications, among others^(^ 14 ^-^ 16 ^)^. The nursing team working in the PACU therefore needs to consider thirst as an object of care intentionally, identifying, measuring, assessing safety and using effective strategies to reduce the child’s thirst^(^ 17 ^)^. The team, however, usually feels insecure to treat thirst^(^ 18 ^)^ in the anesthetic recovery phase, as it does not have systematic instruments that assess safety to offer a method of relieving pediatric thirst, prolonging the suffering of the child and his family^(^ 9 ^,^ 19 ^)^.

To support the team in the decision to use a thirst relief strategy, the Safety Protocol for Thirst Management (SPTM) of adult patients in PACU was elaborated^(^ 20 ^)^. The team has also used this instrument for the infant patient, even without proving that the proposed evaluation criteria for the adult are also relevant for the child.

The instrument validation process is essential for the results to be significant, reliable, precise and accurate^(^ 21 ^)^. Validity and reliability are the main aspects in the process. Validity verifies whether the instrument measures exactly what it proposes to measure and reliability represents the degree of coherence with which the instrument measures the attribute^(^ 22 ^)^.

The need to develop and validate a safety protocol for the management of thirst in children in the IPP is justified by its high prevalence and intensity^(^ 1 ^,^ 23 ^)^. In addition, no instrument was found to support the practice of PACU professionals in the assessment of adequate criteria that allow the effective use of effective strategies to relieve the child’s thirst in this period. The objective of this study was, therefore, to elaborate, validate and evaluate the reliability of the Safety Protocol for Pediatric Thirst Management (SPPTM) in the IPP.

## Method

**Figure 1:**
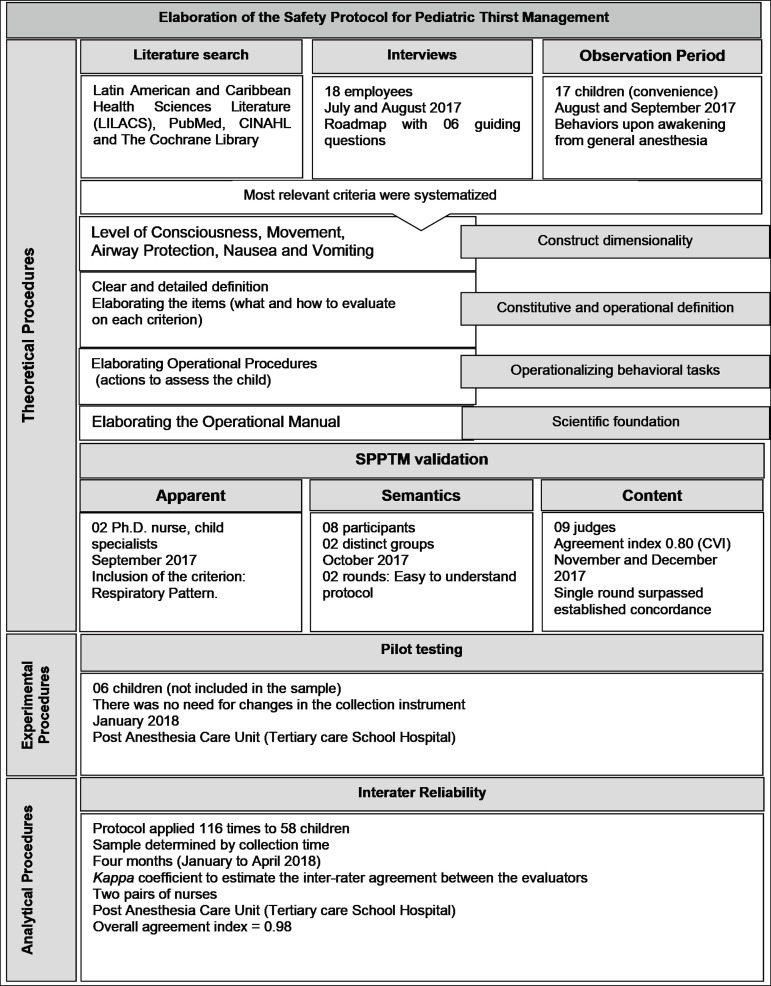
Sequential diagram of the elaboration, validation and reliability of the Safety Protocol for Pediatric Thirst Management

Methodological, quantitative research, carried out between July 2017 and April 2018. In view of the difficulty in finding specific methodologies for the elaboration of protocols that presuppose decision-making for care and aiming to follow a rigorous methodological process, an adaptation of the steps of the Pasquali model was used^(^ 24 ^)^. This model is based on psychometry that measures subjective phenomena and was used by another protocol validation study as a guide to its steps^(^ 20 ^)^. This model consists of three procedures - theoretical, experimental and analytical^(^ 24 ^)^, whose steps are summarized in Figure 1.

In the theoretical procedures stage, it is recommended to search the literature, clustering the knowledge of specialists and observation extracted from practical experience^(^ 24 ^)^. The psychological system was defined *as safety for pediatric thirst management in the immediate postoperative period,* and *assessment criteria* as the property of the psychological system (attributes), whose evaluation is the object of this study. The elaboration of the protocol was carefully based on scientific literature, interviews with specialists and systematic observation of the child’s anesthetic recovery^(^ 24 ^)^.

A literature search was conducted in the: Literatura Latino-Americana e do Caribe em Ciências da Saúde (LILACS), Cumulative Index to Nursing and Allied Health Literature (CINAHL), National Library of Medicine (PubMed) e The Cochrane Library, using the following descriptors: child, pre-school, students, hospitalized child, recovery period from anesthesia, post-operative period, caring for the child, thirst, recovery room, scales, respiration, awareness state, cough, general anesthesia, nausea and vomiting, swallowing, postoperative complications, gastrointestinal content, aspiration pneumonia e oral hydration. The criteria for inclusion were the following: Publications in books and articles indexed in the selected databases with descriptors in Portuguese, Spanish and English, from 1960 onwards, since it was the decade when the first descriptions of the child’s arousal upon awakening from general anesthesia were found. Anesthesiology, child development and growth, and surgical child care books were also examined^(^ 25 ^-^ 27 ^)^.

Eighteen experts were consulted under the following inclusion criteria: having experience in assisting hospitalized children or in the IPP, working in large public and/or private hospitals in the city of Londrina. The invitation was made electronically and later the interviews were scheduled in accessible places for the professional. The interviews took place in person, and the professionals answered a script made up by six guiding questions: What should be observed in the emergency of pediatric anesthesia?; What instruments to use to assess the child’s anesthetic recovery?; Which protective reflexes are most important to be evaluated in the child?; How should the assessment of children recovering from anesthesia be? What needs to be considered to manage a thirst relief strategy in children who recover from anesthesia?, and Is there a difference in age? Responses were recorded and tabulated in an Excel 2010^®^ spreadsheet and analyzed according to the frequency of citations.

The main researcher made a period of systematic observation on the children’s anesthetic recovery. During August and September de 2017 the main behaviors presented when they awoke from the general anesthesia were recorded. Seventeen children submitted to general anesthesia and older than three years were evaluated, selected by convenience according to the researcher’s availability during this period.

The results of the literature search were organized in a table listing the main surgical anesthetic complications, scales for assessing the child’s awareness and criteria for allowing early fluid intake in the IPP. In the second column, the responses of the 18 specialists were systematized, considering the criteria considered relevant for the release of oral liquids during the child’s anesthetic recovery. The third column consisted of the main behaviors of the children observed when they woke up from anesthesia. After extensive analysis by the main researchers, the most relevant common criteria were selected among the three stages.

Next, the constructs were defined, which consists of a clear and precise conceptualization of each criterion selected to assess safety for the management of pediatric thirst^(^ 24 ^)^. In the protocol, they are the items to be evaluated and are described in detail below each criterion. For example, what to evaluate in the criterion “level of consciousness”, what to evaluate in “movement” and so on.

Subsequently, the behavioral representation of the constructs was established^(^ 24 ^)^ and the actions that the nurse must take to assess safety for the management of thirst were defined^.^ Finally, the operational manual was prepared, which presents the theoretical basis of the protocol.

Theoretical analysis was performed by specialists through the apparent validation^(^ 22 ^)^, semantic analysis and content validation^(^ 24 ^)^. Two Ph.D. nurses specialized in children were invited to perform the apparent validation in September 2017. The criterion for choosing these professionals was expertise in the pediatric area and in instrument validation, who were not part of the thirst study group. The semantic analysis^(^ 24 ^)^ occurred in a tertiary-level university hospital in northern Paraná, in October 2017. Eight participants were invited, divided into two distinct groups. The first with four Ph.D. nurses with experience in child care in PACU; the second with four students from the last year of the Nursing undergraduate course. The protocol was presented verbally to the two groups in separate meetings, item by item, later on, the participants were asked to reproduce their understanding on the exposed content.

The content validation took place in November and December 2017 through the Delphi Technic^(^ 28 ^)^. Thirteen professionals were invited, two did not accept to participate and two did not return the instruments in the appointed period. Therefore, nine judges participated, including nurses (n = five), anesthesiologists (n = three) and a speech therapist (n = one). There was a concern to include judges from different academic backgrounds so that the contributions to the instrument could include a multiprofessional look. The judges were chosen according to their experience in child care in PACU in different institutional realities, and one, for her experience in validating instruments. The judges worked professionally in Londrina (PR) and São Paulo (SP), and all had postgraduate education, being the doctorate the most frequent (n = four, 45%). Professional experience was over five years for all the specialists. The judges who participated in this stage were present at other moments in elaborating and validating the protocol: Interview stage (n = five) and apparent validation (n=two).

The invitation was made by telephone, informing the research objectives and how participation would be. Upon agreement, they were asked for the email address for subsequent shipment of the validation instruments. In the first contact via e-mail, a letter was sent with the research objectives, validation procedures and invitation to participate as a judge. In the annex, the protocol, four validation instruments, instrument of characterization of the judge and the Free and Informed Consent Form (FICF). The reply to that email was considered acceptance to participate in the survey. The instruments for validation were adapted from other studies^(^ 20 ^,^ 29 ^)^.

The Content Validity Index was used (*Content Validity Index* - CVI), based on the proportion of judges who considered the item valid^(^ 30 ^)^. The CVI was estimated for each protocol evaluation criterion, set of items, operational procedures and operating manual. The individual CVI was calculated from the ratio of the number of specialists who scored three or four for the item on an ordinal scale of one to four (from does not contemplate to contemplate), or on a dichotomous scale (yes and no), by the total number of experts. The total CVI was calculated from the average of the CVIs of the items^(^ 31 ^)^. The minimum agreement established between the judges was 0.80^(^ 24 ^,^ 30 ^)^. Microsoft Office Excel 2010^®^ was used for the calculations.

Four assessment tools were sent to the judges. First one, the judges evaluated the safety criteria according to the requirements: Attributable (reflects quality aspect for nursing care), Accessible (data is accessed quickly, with minimal extra effort and cost), Communicable (the relevance of the measure can be easily communicated and understood), Effective/accurate (measures what it is proposed to measure), Feasible (the measure is applicable) and Objective (the measure allows clear and precise measurement action, without subjective judgment). The judges indicated points ranging from one to four, with one = does not consider security for thirst management; two = unable to contemplate security for thirst management without review; three = includes security for the thirst management, but needs a minimum change; four = includes security for the management of the thirst.

The second assessed the set of items, ticking yes or no on the following requirements: Behavioral (allows clear and precise assessment), Objectivity (allows punctual response), Clarity (spelled out in a clear, simple and unambiguous way), Relevance (evaluates safety for the management of thirst), and Precision (each evaluation item is distinct from the others, do not elicit confused).

The third assessed the operating procedures using the same requirements as instrument two.

The fourth instrument evaluated the validity of the operational manual, indicating yes or no in the requirements of Descriptor (it is clear and objective in what it proposes to measure) and Scientific Basis (it is sufficient to evidence the indicator).

Within the experimental procedures, a pilot test was carried out with six children in January 2018 to adjust the collection procedures. Initially, the researchers evaluated the child 30 minutes after arriving at the PACU, but it was observed that a longer time was needed to start the evaluation, as they were still sleepy, with limitations to participate in the process. Then, the first assessment was established 45 minutes after arrival at the PACU, and the second, 15 minutes after the first. There was no need for changes in the collection instrument. Pilot test participants were not included in the sample.

The analytical procedures consisted of assessing the protocol’s reliability by inter-rater agreement. The *kappa* coefficient was used to estimate the agreement among the evaluators, calculated by the ratio of the proportion of times the observers agreed (corrected by agreement due to chance) to the maximum proportion of times they could agree^(^ 32 ^)^. The determination of the agreement strength of the *kappa* values followed the following recommendation: Less than zero, poor agreement; from zero to 0.20, negligible agreement; 0.21 to 0.40, smooth agreement; 0.41 to 0.60, moderate agreement; from 0.61 a 0.80, substantial agreement, from 0.81 to one, almost perfect agreement^(^ 33 ^)^.

Reliability was assessed in the PACU of a tertiary-level teaching hospital in the State of Paraná, with the participation of two pairs of nurses. The first was made up by the researcher and a resident in perioperative nursing; the second, by the researcher and a nurse from the PACU. The pairs were chosen for their availability to participate in the research and for their experience in child care in the PACU, conditioned they first participated in the training on the SPPTM.

The sample was determined by collection time, totaling four months. The criteria for inclusion were the following: Surgical children aged between three and 12 years old, of both sexes, to be recovering from anesthesia in the PACU, undergoing procedures of any specialty and anesthetic technique, elective or emergency procedures performed from Monday to Friday, from 7:00 am to 7:00 pm, conditioned to availability of the two evaluators. The exclusion criterion was a child with neurological disorders and mental disorders, as they might not be able to express the necessary answers for the assessment. The minimum age for inclusion was three years old, because, from then on, the child is able to speak his own name, name objects, show ability to move, has more precise movements and can handle objects^(^ 34 ^)^.

The pair of nurses applied the protocol independently and simultaneously, without communication among the evaluators. While one professional applied the SPPTM, the other just followed and recorded the considerations; in the next evaluation, the professionals reversed the order. The pair waited for the child’s arrival at the PACU and the researcher talked to their parents, asking for authorization to carry out the research. At this moment, they were informed on the goals of the study and how the child participation would take place. After having accepted, the primary guardian signed the FICF, the children were asked about their willingness to participate in the research, and no child refused. A 12-year-old child participated in the study and signed the FICF. After arriving at the PACU, the researcher explained the objectives of the research and assessed their intention to take part in it. It was determined that each child could be assessed twice: the first assessment 45 minutes after arriving at the PACU; the second, 15 minutes after the first one. If the child was agitated, tearful or with pain, a longer time was observed to begin collection. One used the program *Statistical Package for Social Sciences* - SPSS^®^ (version 20.0) to calculate the *kappa* coefficient and carry out the descriptive analysis.

## Results

In the literature search, a specific evaluation scale was found when the child awakens from sedation and regains consciousness, with the following items: eye response, appearance and function, and body movement^(^ 35 ^)^. Regarding the main complications in the IPP, pain, nausea, vomiting and emergency delirium (ED) stand out. It is a common condition in children in the IPP, defined as a disturbance in the child’s awareness and attention to his environment, with disorientation and perceptual changes^(^ 12 ^)^, with the presence of restlessness, crying, moaning or irritating speech and screams^(^ 36 ^)^. Few evaluation criteria were found for early fluid release: Spontaneous verbalization of the child^(^ 8 ^-^ 9 ^)^, appears to be awake enough^(^ 8 ^)^ and receive a score that is greater than or equal to four^(^ 9 ^)^ on the *Face, Legs, Activity, Cry, Consolability scale (FLACC),* a scale that assesses the child’s pain.

In the interview stage, the experts pointed out the following safety criteria: assessment of level of consciousness (n = 12), airway protection reflexes (cough n = 17, swallowing n = 12, crying n = three), absence of nausea and vomiting (n = three), movement evaluation (n = five), consider the participation of the main caregiver (n = four), child’s will (n = nine), medical criterion (n = two), surgical time and size (n = one).

During the observation period of the child’s recovery, the main findings were the following: Variability in the time of emergence of anesthesia, bodily behaviors such as movement of limbs and eyes, type of verbalization and presence of crying.

Based on the analysis of the previous steps, the following evaluation criteria were then selected to compose the SPPTM: level of consciousness, movement, airway protection (coughing and swallowing) and absence of nausea and vomiting. The selection sought to meet the maximum requirements for safety, simplicity and ease of application in clinical practice. Then, it was defined with the consulted specialists that the protocol can be used in children aged between three and 12 years old.

**Figure 2:**
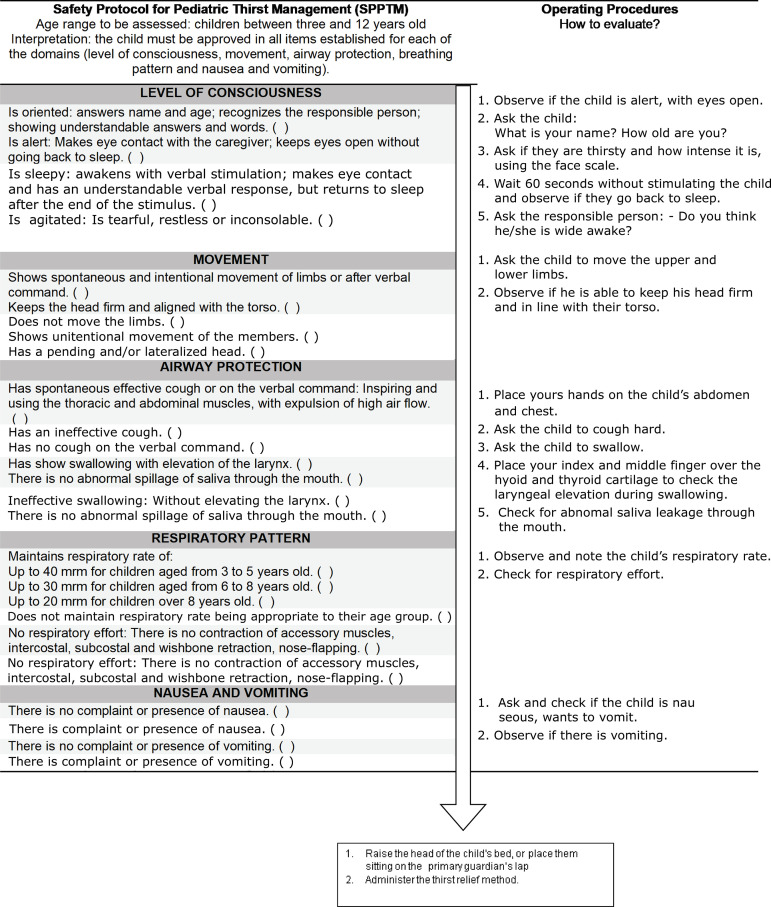
Safety Protocol for Pediatric Thirst Management

Apparent validation resulted in the inclusion of the respiratory standard assessment criteria, which had not been proposed. In its final version, the protocol consisted of five evaluation criteria, arranged in a graphic algorithm (Figure 2), in which it is necessary to approve the child in all the evaluated criteria. The identification of any clinical condition that shows failure in the evaluated criterion represents interruption in the use of the protocol. Then, a new assessment should be started after a period that allows a change in the child’s clinical status.

The five evaluation criteria are described in capital letters in the protocol and identified in dark gray. Below each one of them there are the items to be evaluated and in the right column are the operational procedures. The sentences highlighted in light gray represent approval in the criterion. Additionally, the operational manual was elaborated, which contains the theoretical basis for the protocol. The manual can be obtained in full in the author’s master’s dissertation.

As for the semantic analysis, the group of students and the group of Ph.D. nurses did not have any difficulty to understand the items. They only made some editorial adjustments and changes to the evaluation orders.

A single round of content evaluation by the experts was sufficient to overcome the minimum agreement of 0.80^(^ 24 ^,^ 30 ^)^. Table 1 shows the CVI values of the evaluation criteria and their representative items.

**Table 1:** Validation of protocol content in relation to the evaluation criteria and their representative items. Londrina, PR, Brazil, 2017 (n = 9)

Review Criterion	CVI
Level of Consciousness	1
Oriented	0.91
Alert	1
Sleepy	1
Agitated	1
Movement	0.96
Spontaneous and intentional movement	0.93
Head firm and aligned to the torso	0.84
They do not move the limbs	0.84
Unintentional movement	0.95
Head hanging and/or lateralized	0.97
Airway Protection	1
Effective cough	0.97
Ineffective cough	1
No cough	0.97
Deglutition with elevation of the larynx	1
No abnormal spillage of saliva through the mouth	1
Ineffective swallowing	1
Abnormal saliva leakage through the mouth	0.97
Respiratory Pattern	1
Respiratory rate appropriate to your age group	0.97
Respiratory rate inappropriate for their age group	1
No respiratory effort	0.93
Respiratory effort	0.93
Nausea and Vomiting	1
There is no complaint or presence of nausea	0.93
There is complaint or presence of nausea	0.93
There is no complaint or presence of vomiting	0.93
There is complaint or presence of vomiting	0.95
Total CVI of the criteria*	0.99
Total CVI of the items^†^	0.95

* CVI = *Content Validity Index* total criteria - obtained through the mean (sum of the CVI of each criterion divided by the total number of criteria);

† *Content Validity Index* (CVI) total of items - obtained through the average (sum of the CVI values of each item divided by the total number of items)

Table 2 displays the CVI values of the operating procedures and the operating manual for each criterion.

**Table 2:** Validation of protocol content in relation to the operational procedures and operational manual of each evaluation criterion. Londrina, PR, Brazil, 2017 (n = 9)

Evaluation Criterion	CVI	CVI
	Operating Procedures	Operative Manual
**Level of Consciousness**	0.95	0.94
	(1) Observe if the child is alert, with eyes open. (2) Ask the child: What is your name? What is your age? (3) Ask if he is thirsty and how intense it is, using the face scale. (4) Wait 60 seconds without stimulating the child and observe whether he goes back to sleep. (5) Ask the primary guardian: Do you think he/she is wide awake?	
**Movement**	0.97	0.88
	(1) Ask the child to move the upper and lower limbs. (2) Observe if he is able to keep his head steady and in line with his torso.	
**Airway Protection**	0.91	0.94
	(1) Place your hands on the child’s abdomen and chest. (2) Ask the child to cough hard. (3) Ask the child to swallow. (4) Place the index and middle finger on the hyoid and thyroid cartilage to check the laryngeal elevation during swallowing. (5) Check for abnormal saliva leakage through the mouth.	
**Respiratory Pattern**	1	1
	(1) Observe and note the child’s respiratory rate. (2) Check for respiratory effort.	
**Nausea and Vomiting**	0.93 (1) Ask and check if the child is nauseous, wants to vomit. (2) Observe if there is vomiting.	0.94
Total CVI of operating procedures*	0.95	
**CVI of the operating manual^†^**	0.94	

* *Content Validity Index* (CVI) total of operational procedures - obtained by means of the average (sum of the CVI of each set of operational procedures of the evaluation criteria divided by the total number of criteria);

† *Content Validity Index* (CVI) total of the operating manual - obtained through the average (sum of the CVI of the operating manual of each evaluation criterion divided by the total number of criteria)

During the reliability assessment, SPPTM was applied 116 times in 58 children. The mean age was 7.2 years old (sd 2.6). Children of all ages for whom the protocol was developed took part: three years (n = seven), four years (n = three), five years (n = eight), six years (n = seven), seven years (n = six), eight years (n = six), nine (n = seven), ten years (n = five), 11 years (n = eight), and 12 years (n = one).

Male children predominated 42 (73%); the frequency of procedures by surgical clinics was: infant and pediatric surgery 32 (55%), otorhinolaryngology 15 (26%), orthopedics eight (14%), ophthalmology two (3%), head and neck one (2%). The anesthetic technique with the greatest use was general anesthesia 51 (88%). As for the classification of surgical risk, according to ASA, most were classified as ASA I, 49 (84%), followed by classification II, nine (16%). The majority of the procedures was of elective nature 50 (86%), being eight (14%) of urgency.

Table 3 shows the values of *kappa* calculated for each SPPTM evaluation item, with almost perfect agreement for all items^(^ 33 ^)^.

**Table 3:** Kappa coefficient of the items of the Safety Protocol for Pediatric Thirst Management evaluated by nurses. Londrina, PR, Brazil, 2018 (n = 58)

Evaluation Criterion	Agreement Percentage	Kappa coefficient*
Level of Consciousness		
Is oriented	99.1	0.96
Is alert	96.6	0.89
Is sleepy	96.6	0.89
Is agitated	100	1
Movement		
Spontaneous movement	100	1
Head firm and aligned to the torso	100	1
Do not move the limbs	100	1
Unintentional movement	100	1
Head hanging and/or lateralized	100	1
Airway Protection		
Effective and spontaneous cough	100	1
Ineffective cough	100	1
Has no cough	100	1
Ineffective swallowing	100	1
There is no abnormal spillage of saliva	100	1
Ineffective swallowing	100	1
There is no abnormal spillage of saliva	100	1
Respiratory Pattern		
Adequate respiratory rate	100	1
Inadequate respiratory rate	100	1
No respiratory effort	100	1
Respiratory effort	100	1
Nausea and Vomiting		
There is no complaint or presence of nausea	100	1
There is complaint or presence of nausea	100	1
There is no complaint or presence of vomiting	100	1
There is complaint or presence of vomiting	100	1
		
***Kappa* total^†^**		**0.98**

* *Kappa coefficient*: less than zero, poor agreement; from zero to 0.20, negligible agreement; 0.21 to 0.40, smooth agreement; 0.41 to 0.60, moderate agreement; 0.61 to 0.80, substantial agreement, 0.81 to 1, almost perfect agreement^(^ 20 ^)^

† *Kappa* total realized through the average of individual items values

## Discussion

The contribution of this study consists of making available an unprecedented, judicious, objective, valid and accurate instrument that allows assessing safety to manage strategies for relieving thirst for infant patients in the IPP. For the elaboration, validation and evaluation of the protocol’s reliability, high scientific rigor followed^(^ 24 ^)^.

The interviews with specialists made it possible to observe how diverse and subjective the criteria used by the professionals responsible for authorizing methods to relieve thirst in the IPP are. Professionals reported that, most of the time, they look at the child in the PACU and assess whether, apparently, they are awake enough and without complaints, then they allow the intake of liquid orally. However, this assessment is not standardized or based on criteria and varies according to the determination of “being well awake” by each professional. It was also observed that, when liquid intake is authorized, there is no consensus as to the type and volume to offer. There were reports on the limitation of specific literature for the child, resulting in adapted evaluations, which consider criteria of adult patients. Currently, the anesthesiologist is responsible for the authorization for liquid oral ingest in the PACU, which explains the greater number of them in the interview stage.

The “level of consciousness” criterion was one of the most frequently suggested by professionals, considered an essential item to determine the emergence of the anesthetic state during the IPP. When asked about the scales used to assess children’s awareness, the answers were varied: Glasgow comma scale^(^ 37 ^)^, *Comfort-Behavior* ^(^ 38 ^)^, Index Steward^(^ 39 ^)^ scale of Aldrete and Kroulik^(^ 40 ^)^. However, the Glasgow and Comfort-B scales do not apply to children in the IPP, because they assess the level of sedation and have been validated for children in the intensive care unit. The Steward Index^(^ 39 ^)^ and the scale of Aldrete and Kroulik^(^ 40 ^)^, although targeted at patients in the PACU, may not be adequate to be used with a child^(^ 35 ^)^.

A scale for assessing the child’s consciousness after sedation was found in the literature ^(^ 35 ^)^. This is the *Vancouver Sedative Recovery Scale* (VSRS), a scale made up by 12 items covering three categories of indicators: Response, appearance and function of the eyes, and body movement. Reliability was assessed in 82 children aged between nine months and 17 years old. The internal consistency measured by Cronbach’s alpha was 0.85, interobserver agreement 0.90, and values of *kappa* for the individual items ranged from 0.65 to 0.89^(^ 35 ^)^, values similar to those found in this study. Some items on this scale are similar to those of the SPPTM: The child is alert, sleepy, able to make eye contact, presence of spontaneous and intentional movements.

The other scales found in the literature consist of ED measurement scales. One of the most used scales to measure this condition is the Pediatric Anesthesia Emergence Delirium (PAED), made up by the following items: the child makes eye contact with the caregiver; the child’s actions are purposeful; the child is aware of the surroundings; the child is restless, and the child is inconsolable. This scale was evaluated on 46 children aged between 18 months and six years and displayed an internal consistency of 0.89 and a reliability of 0.84^(^ 12 ^)^. Therefore, for selecting the items for evaluating the SPPTM awareness level criterion, the presence of these behaviors was considered.

When evaluating the item “is oriented” in the behavioral requirement, some experts indicated that children aged between three and five years could possibly not answer their name and age because they are in an unknown environment and regaining consciousness. There was no such difficulty during the application of the protocol in practice. However, this study employed a convenience sample, and a larger number of this population would be needed to assess this issue in depth.

It is more difficult to assess the child’s level of consciousness than that of the adult, and it is challenging to identify the child’s inability to communicate^(^ 35 ^)^. When assessing reliability, the evaluators disagreed on the items “is alert” and “is sleepy”, confirming the difficulty and subjectivity in assessing the child’s level of consciousness. The need for a period of interaction with the child was identified before starting up the assessment.

Two judges considered the criterion “movement” as not relevant in measuring safety for the thirst management. For others (n=three), it represents an evaluation criterion complementary to the level of consciousness, measured by the ability to perform intentional movements and keep the head firm and aligned with the trunk. Additionally, the presence of voluntary and purposeful movements is part of the scales for assessing the child’s consciousness^(^ 12 ^,^ 35 ^)^, justifying the choice to keep this item in the protocol. In addition, the ability to move with intentionality may indicate reversal of general inhaled anesthetics and neuromuscular blockers.

The evaluation of criterion “airway protection” ensures the verification of the return of protective cough and swallowing reflexes. These reflexes indicate that the patient is able to defend himself against a possible bronchopulmonary aspiration^(^ 41 ^)^. The incidence of perioperative pulmonary aspiration in pediatric patients varies from one to ten in 10,000. Additionally, when there is a consequence, it is considered mild and, to date, there have been no reports of mortality from pulmonary aspiration in children^(^ 4 ^)^. Evaluating the protective reflexes in the SPPTM presupposes the evaluation of cough and swallowing.

Two experts pointed out in the content validation that the assessment of protective reflexes (coughing and swallowing) could encounter some difficulty with younger children. However, they considered this item as of extreme relevance in order to determine the safety for oral liquid release in the IPP. Therefore, a prior approach to the child is recommended, in order to reduce the anxiety and fear present in this period, so that there is a bond and trust in the moment of assessment.

During interviews with specialists, it was mentioned that crying could be considered a protective reflex, indicating that the child’s airway would be free. But crying can represent several situations, such as pain, discomfort, irritation, agitation and ED. Differentiating their presence is difficult and subjective, therefore, in the protocol, the presence of crying characterizes the child’s failure to receive a method of relieving thirst.

The “breathing pattern” consists of the assessment of respiratory frequency and respiratory effort, when signs of accessory muscle contraction, intercostal, subcostal and wishbone retraction, and nose wing beats must be absent^(^ 42 ^)^. For some professionals, the evaluation of this criterion signals the main changes in the child’s clinical status. Furthermore, adverse perioperative respiratory events represent one of the main reasons for morbidity and mortality in children^(^ 43 ^)^.

The absence of “nausea and vomiting” is paramount for administering methods for relieving thirst. The presence of vomiting is still a complication feared by the team due to the possibility of subsequent pulmonary aspiration, although recently, its incidence is between 25% and 30% in children undergoing general anesthesia^(^ 44 ^)^. The absence of these complications indicates reversion of anesthetic agents.

Clinical trials have evaluated whether post-operative fasting would reduce the incidence of nausea and vomiting in children. One study found no statistically significant difference between the two groups observed, with incidence of 15% in the liberal group and 22% in the fasting group (p = 0.39)^(^ 8 ^)^. Another study revealed an association between early postoperative oral fluid intake and a reduction in the incidence of vomiting, which was 11.4% in the liberal group and 23.9% in the fasting group^(^ 9 ^)^. In both studies cited, the child’s willingness to receive liquid and food was considered. When the child is forced to drink fluid early, there is increased vomiting incidence^(^ 45 ^)^. The experts considered the child’s willingness to drink and the child’s verbalization as relevant evaluation criteria. Therefore, when questioning the presence of thirst in the child, it is also necessary to question his willingness to receive any strategy to relieve thirst and only then begin the SPPTM assessment.

The application of SPPTM by nurses showed a high overall value of the *kappa* coefficient. This means that this instrument has inter-rater agreement, indicating that it can be reproduced in other realities. Thus, there is an indication that this instrument is a useful tool for the nursing care in the PACU, minimizing the presence of a prevalent and intense symptom such as thirst, especially for the infant patients.

One of the obstacles encountered in conducting this study was the scarcity of instruments to assess the child’s anesthetic recovery, resulting in the difficulty of structuring the criteria to direct the child’s assessment in this period in relation to the release of liquid orally by the professionals. This study, therefore, has come to fill a gap in the literature and to subsidize the care provided to the surgical child with thirst.

Assessing safety for thirst management, using relevant selected criteria, allows nurses to look intentionally at a frequent symptom and to safely intervene safely in its management. It is noteworthy that the protocol was designed for children who do not have communication limitations and children without contraindications to receiving oral fluids in the IPP.

The limitation of this study was centered on the convenience sample. It is suggested, therefore, that the protocol be applied to a larger number of children, in other institutions and with stratification by age. Further studies are needed to assess factors associated with approval of the protocol, as well as the most suitable moments for its use in the child’s anesthetic recovery. Even so, the reliability values of the SPPTM were high, indicating the accuracy of this instrument.

## Conclusion

The SPPTM was elaborated based on the relevant signs and symptoms in determining safety for administering methods to relieve pediatric thirst in the IPP. The safety criteria and their representative items were identified after a rigorous scientific basis, interviews with specialists and a period of systematic observation of the child’s anesthetic recovery.

This unprecedented protocol proposes five evaluation criteria: Level of consciousness, movement, airway protection (coughing and swallowing), breathing pattern (respiratory rate and respiratory effort), and nausea and vomiting.

The judges performed theoretical analyzes through apparent, semantic and content validity. The SPPTM is easy to understand, has relevant and relevant content, with a high level of agreement among the judges on all the items evaluated. This indicates that the evaluation criteria proposed by the protocol measure with satisfaction the safety for the management of pediatric thirst.

When evaluating the reliability of the protocol in its practical application with surgical children aged between 3 and 12 years in the IPP, it was possible to observe an almost perfect agreement between the evaluators.

The SPPTM is, therefore, a valid and accurate instrument, indicating that it is a useful tool for use in clinical practice in the PACU, enabling the safe management of pediatric thirst.
